# Role of micro-RNAs 21, 124 and other novel biomarkers in distinguishing between group 1 WHO pulmonary hypertension and group 2, 3 WHO pulmonary hypertension

**DOI:** 10.1186/s43044-023-00395-w

**Published:** 2023-08-30

**Authors:** Mark O. Dimitry, Youssef M. A. Soliman, Reem I. ElKorashy, Hala M. Raslan, Solaf A. Kamel, Eman M. Hassan, Fatma Elzahraa Ahmed, Rasha N. Yousef, Eman A. Awadallah

**Affiliations:** 1 Cardiology Unit, Department of Internal Medicine, National Research Center, Cairo, Egypt; 2 Pulmonary Vascular Disease Unit, Department of Pulmonology, Cairo University, Cairo, Egypt; 3 Department of Clinical and Chemical Pathology, National Research Center, Cairo, Egypt

## Abstract

**Background:**

Pulmonary hypertension “PH” is considered a serious cardiovascular disease. World Health Organization divided PH into groups depending on many factors like pathological, hemodynamic, and clinical pictures. Lately, various micro-RNAs “miRNAs” and other novel biomarkers like endoglin and asymmetric dimethylarginine “ADMA” might have a role in diagnosis of PH and may differentiate between pulmonary arterial hypertension “PAH” and non-PAH. The purpose of the study is to show the role of miR-21, miR-124, endoglin and ADMA in the diagnosis of PH and distinguishing between WHO group 1 PH and WHO group 2 and 3 PH and to identify patients who might benefit from non-invasive and inexpensive tools to diagnose PAH.

**Results:**

miR-21 was upregulated in group 1 PH, and there was significant difference between group 1 PH as compared with group 2 PH, group 3 PH and control; miR-124 was down-regulated in group 1 PH with highly significant difference between group 1 and group 2 PH and control but no significant difference with group 3 PH, endoglin was elevated in group 1 PH with a significant difference as compared to group 2 PH, group 3 PH and control. ADMA was elevated in group 1 PH as compared to control; however, there was no significant difference between it and group 2, 3 PH.

**Conclusions:**

miR-21, miR-124, endoglin and ADMA are good biomarkers to diagnose PH; however, only miR-21 and endoglin could distinguish group 1 PH from group 2 and 3 PH.

## Background

Pulmonary hypertension “PH” is considered a serious cardiovascular disease. World Health Organization “WHO” divided PH into groups depending on many factors like pathological, hemodynamic and clinical pictures [1]]. PH is now managed with better diagnostic and treatment tools as compared with the past [2, 3].

Group 1 PH is diagnosed as a mean pulmonary artery pressure “mPAP” ≥ 20 mmhg at rest with pulmonary capillary wedge pressure “PCWP” ≤ 15 mmHg and pulmonary vascular pressure “PVR” > 2 Wood units “WU” [4].

Left heart diseases are one of the most causes of group 2 PH [5]. It is diagnosed as a PAPm of ≥ 20 mm Hg plus a PCWP or left ventricular end-diastolic pressure “LVEDP” ≥ 15 mm Hg. The commonest causes of group 3 PH are chronic obstructive pulmonary disease “COPD,” interstitial lung disease “ILD” and obstructive apnea. The incidence of PH in COPD and ILD is high and reach more than 50% in some studies [6].

One of the most challenging issues is the distinguishing between group 1 PH and other groups especially group 3 PH especially when there are no specific changes differentiating group 1 PH from other forms of PH. Diagnosis of group 1 PH may be strongly accepted if PAH is caused by a known mutation or there is a strong family history of group 1 PH; otherwise, expensive diagnostic tools might be needed to diagnose group 1 PH. So, there is a need for biomarkers allowing differentiating between group 1 PH and PH due to other causes especially chronic lung disease. [7].

MiRNAs have been used recently as markers that can be used for diagnosis and follow-up of the PH progress [8]. The gene expression was regulated negatively by the intracellularly miRNAs across the transcriptome by involvement of specific nucleic acid sequences which leads protein translation inhibition. The pathogenic consequences in PH are affected by the dynamic alteration in their expression, and so might be of diagnostic or prognostic role [9]. MiRNAs can affect several biological processes like cell survival, proliferation and differentiation [10].

ADMA and endoglin help in the diagnosis of PH. ADMA is a natural amino which inhibits the production of nitric oxide “NO.” The release of NO is decreased by the high concentration of ADMA which affects the pathway of NO/cGMP, and this will lead to increase in vascular tone. [11]. ADMA plasma levels might be helpful in monitoring the PAH treatment [12].

Endoglin which was assumed to regulate angiogenesis is considered an important biomarker for diagnosis of patients with PAH and its expression in microvascular endothelium was significantly enhanced [13].

### Purpose of the study

- To assess the role of miR-21, miR-124, endoglin and ADMA in the diagnosis of PH.
- To develop non-invasive and inexpensive tools that can distinguish between group1 WHO from PH secondary to left sided heart disease “group 2 WHO PH” or lung diseases “group 3 WHO PH.”

## Methods

Eighty-eight subjects were included in the study. They were enrolled from cardiology clinic of medical services unit at National Research Center and pulmonary vascular disease unit in chest hospital of Cairo University.

The subjects were divided into 4 groups:

1. 30 patients diagnosed with group 1 PH with age range from 20 to 62 years, 10 males, 20 females
2. 20 patients diagnosed with group 2 PH with age range from 36 to 67 years, 11 males, 9 females
3. 18 patients diagnosed with group 3 PH with age range from 32 to 69 years, 12 males, 6 females
4. 20 patients apparently healthy volunteers as controls with age range from 22 to 60 years, 10 males, 10 females

All patients were documented to have PH by right heart catheterization for group 1 PH and other non-invasive tools (echocardiography, chest X ray and CT chest) for group 2, 3 PH. We excluded patients with group 4 and 5 PH, patients with uncertain diagnosis, patients with end stage PH, patients with malignancy or any other systemic diseases.

All the studied groups were subjected to:

1. Full medical history taking with special emphasis on symptoms of PH, drug intake and family history of PH.
2. Full clinical examination including cardiac and chest examination
3. Right heart catheterization
  RHC was done to diagnose patients with group 1 PH. A pulmonary artery catheter was introduced to the superior vena cava, right atrium, right ventricle, and PA by qualified operators with patients in the supine position after proper catheter calibration. Pressure waveforms from the RA, PA, and PCWP positions were measured at end expiration. Group 1 PH was diagnosed as a PAPm ≥ 20, PCWP ≤ 15 mmHg and a PVR >3 Wood units in the absence of other causes of precapillary PH like lung diseases, CTEPH.
4. Transthoracic echocardiography
  TTE by using Acuson Siemens machine was performed in the left lateral decubitus position in all patients.
  Left side assessment included:
  LV, LA dimensions and functions.
  Regional wall motions.
  Valvular lesions.
  Congenital abnormalities.
  Right sided assessment included:
  RV, RA dimensions and functions.
  TV velocity and right ventricle systolic pressure (RVSP).
  Inferior vena cava (IVC) diameter and collapsibility.
5. Chest X ray:
  Plain chest X ray was done as a work up to diagnose of type 3 PH
6. CT chest:
  Non-contrast high resolution CT chest was done as a work up to diagnose group 3 PH.
7. Laboratory tests
  A blood sample “5 mL” was taken from each subject then divided, processed and stored according to protocol of each test.

Enzyme-linked immunosorbent assay was used to measure ADMA, endoglin levels after applying the instructions on kits purchased from Elabscience “California, USA catalog No. E-EL-0042.” USA.

Enzyme-linked immunosorbent assay after following instructions on kits purchased from catalog No. E-EL-H 6010 United States.

MiRNAs were extracted from plasma according to the serum/plasma miRNeasy kit protocol, catalog number: 217,184 “Qiagen, Hilden, Germany.” All isolated miRNA were quantified using NanoDrop 1000 “Nanodrop, Wilmington, Delaware, USA.” After the extraction of miRNA, cDNA synthesis was performed using miScript II Reverse Transcription Kit “Qiagen, Germany; catalog number: 218161” by adding 4 µl of miScript HiSpec buffer with 2 µl of 10x miScript Nucleics Mix, 7 ul of nuclease free water, 2 ul of miScript II Reverse Transcription mix and 5 ul of the isolated miRNA. The resulting double-stranded “ds” cDNA was a template for an in vitro transcription “IVT” reaction by using SYBER Green PCR Kit “catalog No. 218073”, by adding 12.5 ul master mix, 2.5 ul universal primer, 2.5 ul primer assay, 4.5 ul nuclease free water and 3 ul of cDNA.

Real-time Quantitative PCR was performed using Rotor gene Q Real Time PCR System “Qiagen, Valencia, CA, USA.” The relative expression levels of miR-21, miR-124 were calculated and normalized to miR-16 “Applied Biosystems, Foster City, CA” using 2^−ΔΔct^ method [14]. MiR-16 was used as a standard miRNA to compare expression levels of other miRNAs in serum. The CT is defined as the PCR cycle at which the fluorescent signal of the reporter dye crosses an arbitrarily placed threshold [15].

### Statistical analysis

SPSS version 18.0 for Windows from SPSS, Inc. “IL, USA” was used for data entry and analysis. Qualitative data that presented by numbers and percentages were assessed by using Chi square. Continuous data were expressed as mean and standard deviation. We compare between two means by using *t*-test and more than two means were assessed by using ANOVA. Two or more independent samples were compared by using Kruskal–Wallis tests when the data are not normally distributed.

Receiver-operating characteristic “ROC” analysis was used to obtain the area under the curve “AUC” and the corresponding 95% CI. The maximum cutoff point was measured which corresponds to the highest Youden index for each biomarker. The AUC for each significant biomarker was assessed by using the ROC curve. The sensitivity, specificity, positive predictive values “PPV” and negative predictive values “NPV” were calculated for the identified cutoff points for each biomarker. *P* value *<* 0.05 is considered significant, and < 0.01 is considered highly significant.

## Results

Thirty patients with group 1 PH, 20 patients with group 2 PH and 18 patients with group 3 PH were included in the study. 20 apparently healthy volunteers as control group were also included in the study.

- The results showed no significant difference between the 3 groups of patients and the controls as regards age, sex and Body mass index “BMI” (Table 1)

**Table 1:** Comparison between the 3 groups of PH and controls regarding age, sex and BMI

		Group I	Group II	Group III	Control group	*P* value
		No. = 30	No. = 20	No. = 18	No. = 20	
Age	Mean ± SD	39.17 ± 11.44	45.95 ± 8.99	45.0 ± 6.82	43.85 ± 10.56	0.070
Sex	Females	20 (66.7%)	9 (45.0%)	6 (33.3%)	10 (50.0%)	0.081
	Males	10 (33.3%)	11 (55.0%)	12 (66.6%)	10 (50.0%)	
BMI	Mean ± SD	28.6± 5.9	31.5± 7.2	29.7 ± 5.1	28.1 ±4.8	0.085

**Fig. 1:**
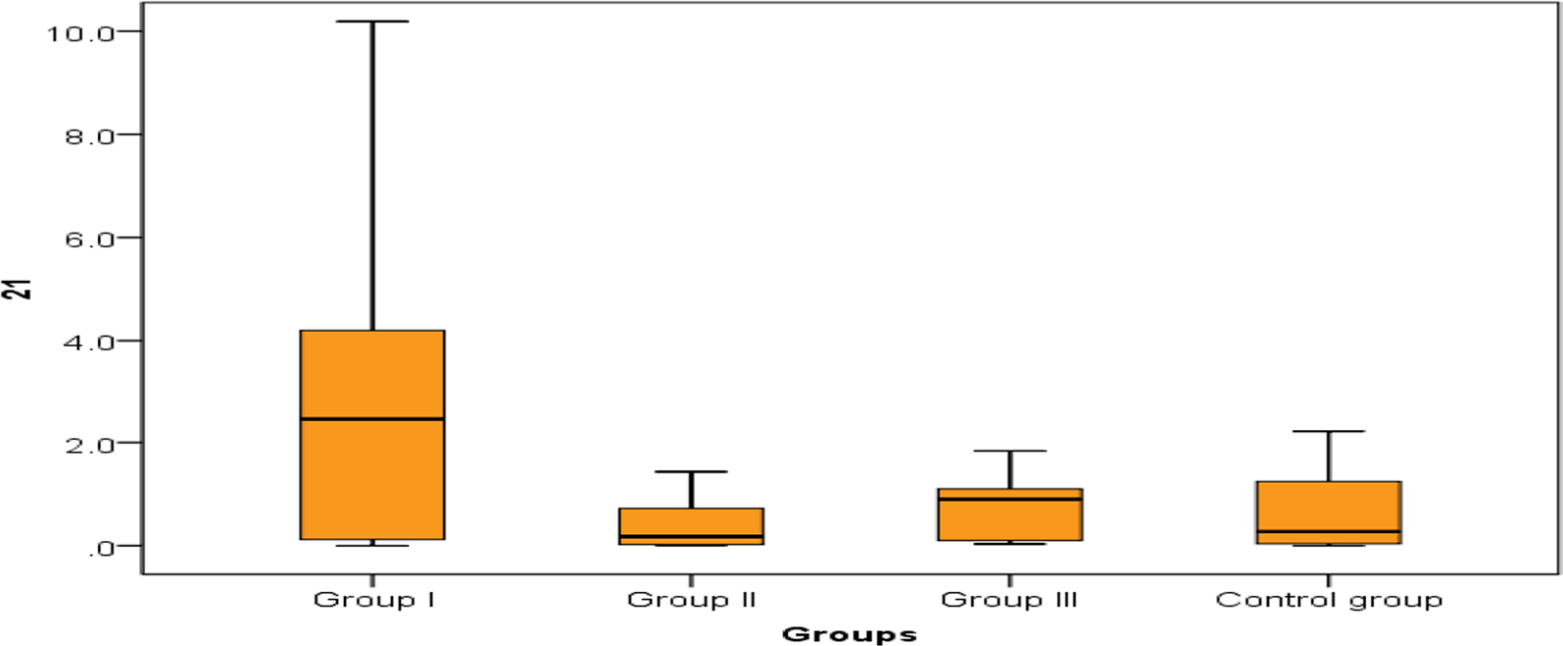
Comparison between the studied groups regarding miR-21

**Fig. 2:**
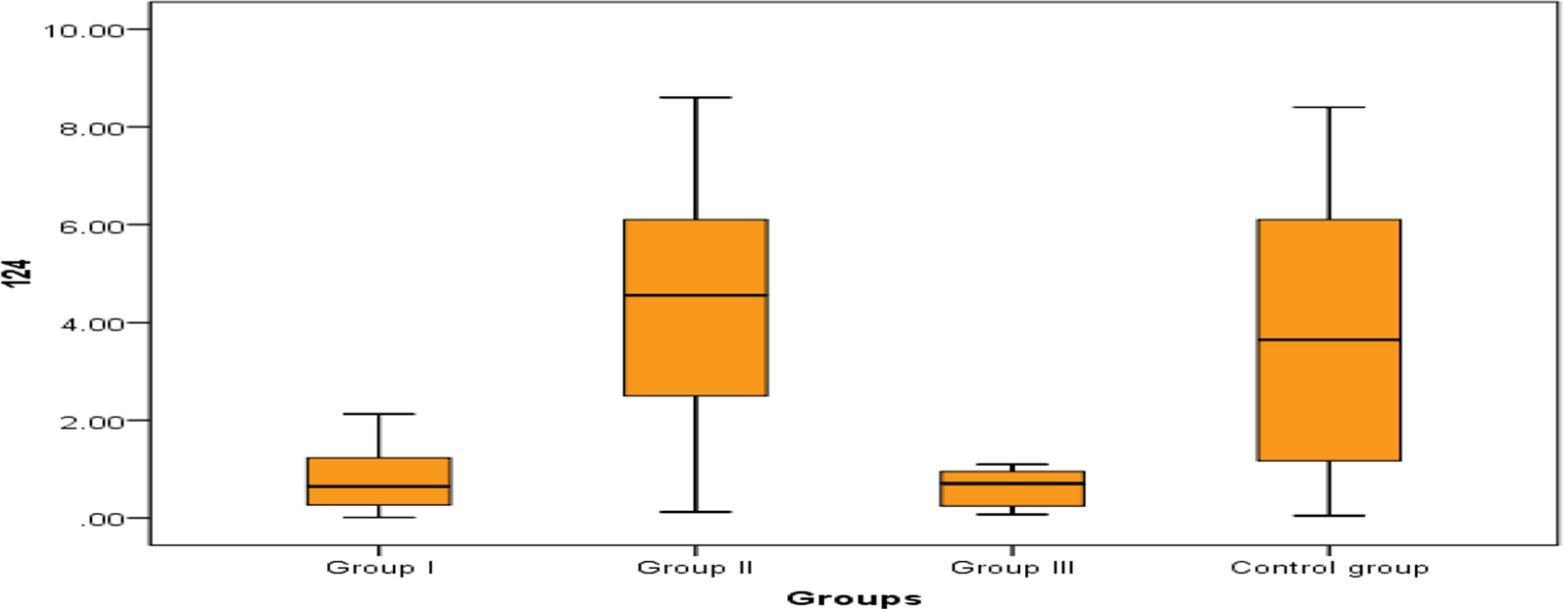
Comparison between the studied groups regarding miR-124
- Regarding the potential value of ADMA and endoglin to differentiate between group 1 PH and group 2, 3 PH and control, we found no significant difference as regard serum ADMA levels between the 3 groups of the patients; however, it was significantly elevated in group 1, 2 and 3 PH patients as compared to controls which indicate that ADMA could be helpful in the diagnosis of PH in general but couldn't differentiate between the PH groups. On the other hand, serum endoglin levels were significantly higher in group 1 PH compared to group 2 and group 3 PH and to controls (Table 3, Figs. 3, 4).

*P* > 0.05 is considered non-significant
- Plasma expression of miR-21 was significantly higher among patients with group 1 PH compared to patients with group 2 and group 3 PH and controls, no significant difference between group 2 PH, group 3 PH and controls, which indicates that miR-21 could be a reliable biomarker differentiating group 1 PH from other 2 groups of PH. As regards plasma expression of MiR-124, it was lower in group 1 PH patients and group 3 PH patients as compared to group 2 PH patients and controls, indicating the value of miR-124 to differentiate group 1 PH from group 2 PH and control. Both miRNAs could be used as diagnostic biomarkers for group 1 PH (Table 2, Figs. 1, 2).

**Table 2:** Comparison between the studied groups regarding miR-21 and miR-124 expression

		Group I	Group II	Group III	Control group	*P* value
		No. = 30	No. = 20	No. = 18	No. = 20	
MiR-21	Median (IQR)	2.45 (0.12–4.2)	0.18 (0.02–0.73)*	0.9 (0.1–1.1)**	0.27 (0.04–1.25)***	< 0.001
	Range	0–3	0.01–1.43	0.04–1.84	0–2.22	
MiR- 124	Median (IQR)	0.65 (0.27–1.23)	4.55^a^ (2.5–6.1)	0.71 (0.25–0.95)	3.65^a^ (1.17–6.1)	< 0.001
	Range	(0.01–2.99)	(0.12–8.6)	(0.07–1.1	0.5–8.4)	

*Highly significant difference with group 1 (*P* = 0.005)

**Significant difference with group 1 (*P* = 0.043)

***Highly significant difference with group 1 (*P* = 0.004)

^a^Highly significant difference with group 1 (*P* < 0001)

**Table 3:** Comparison between the studied groups regarding ADMA and endoglin

		Group I	Group II	Group III	Control group	*P* value
		No = 30	No = 20	No = 18	No = 20	
ADMA	Median (IQR)	4.26 (3.99–4.69)	4.14 (3.78–4.48)	4.15 (3.87–4.32)	3.15 (2.8–3.7)^a^	< 0.001
	Range	3.2–4.82	3.13–4.78	2.33–4.45	2.06–3.81	
Endoglin	Median (IQR)	3.75 (2.9–4.6)	1.6 (0.7–2.6)*	2.6 (2.1–3.3)**	0.55 (0.15–1.05)*	< 0.001
	Range	1.2–8.7	0.1–5.1	1–6	0.1–6.1	

^a^Highly significant difference with group 1, 2 and 3 (*P* < 0.001)

*Highly significant difference with group 1(*P* < 0.001)

**Significant difference with group 1 (*P* = 0.011)

**Fig. 3:**
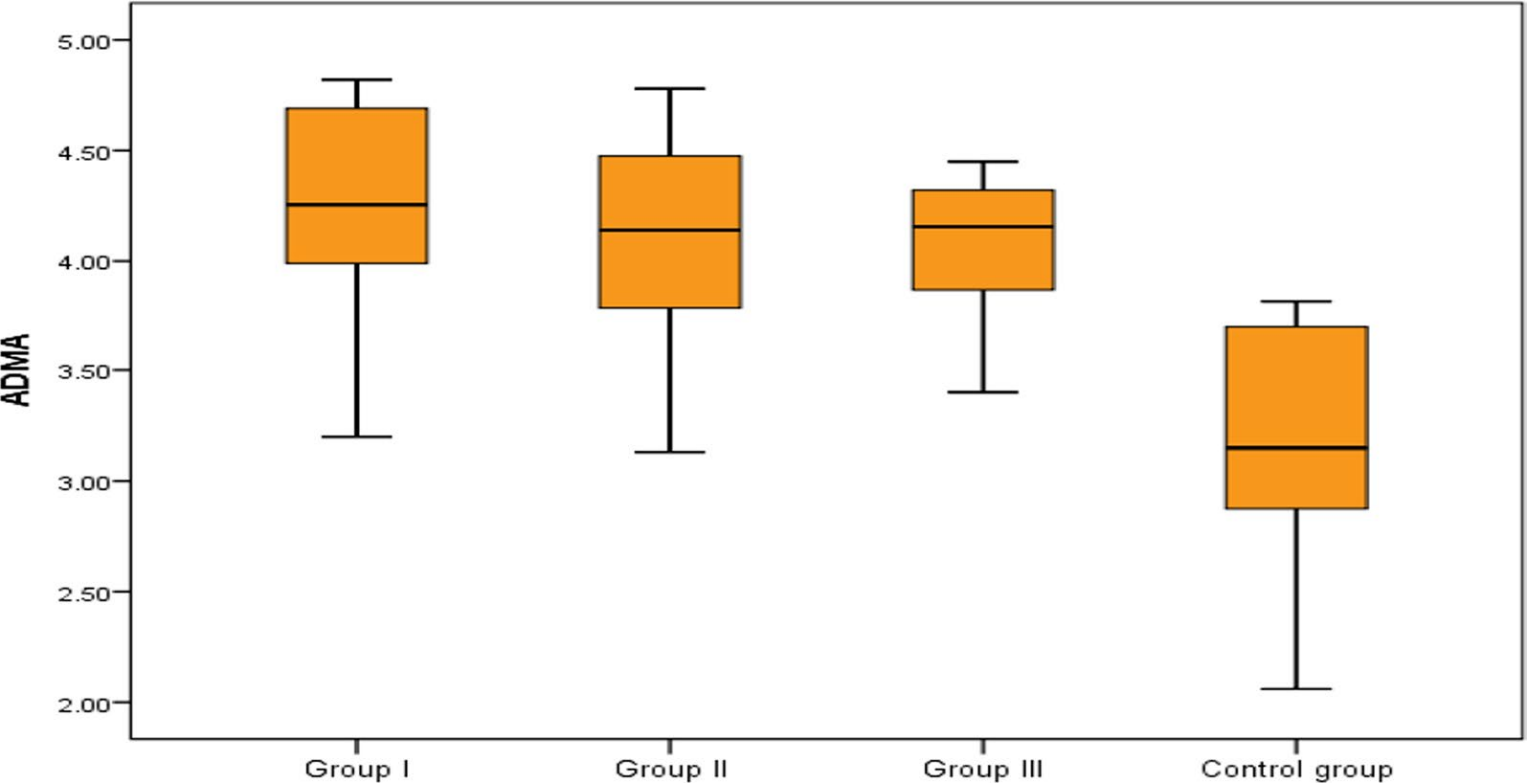
Comparison between the studied groups regarding ADMA

**Fig. 4:**
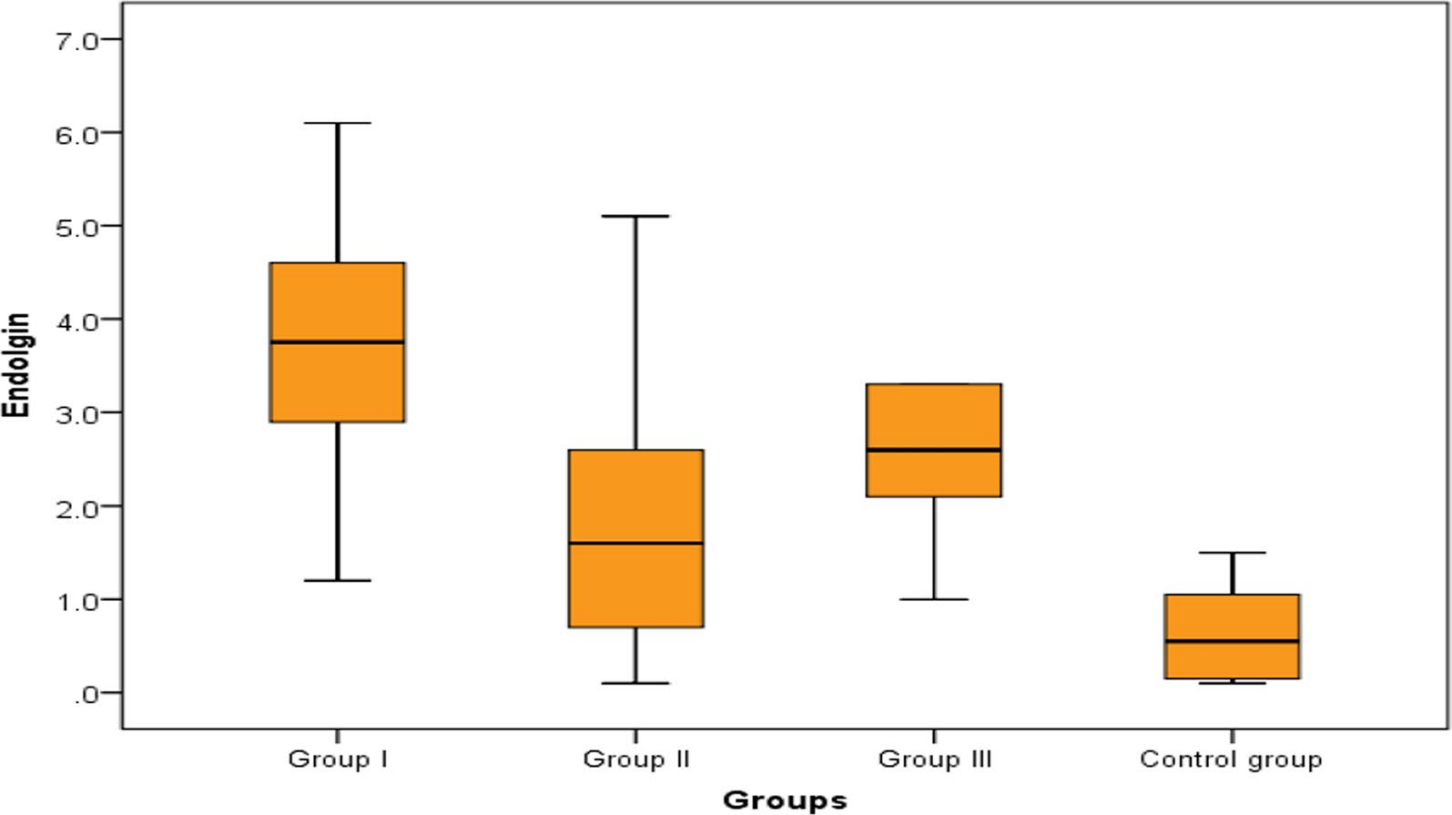
Comparison between the studied groups regarding endoglin
- To assess predictive value of miR-21, miR-124 and endoglin in the diagnosis of group 1 PH constructed, we constructed ROC curve. We found that the cut off predictive value of miR-21 was ≥ 1.55 µM with 60% sensitivity, 95% specificity with AUC (0.742). The cut off predictive value of miR-124 was ≤ 1.58 µM with 86.7% sensitivity, 75% specificity with AUC (0.789). As regard endolgin, the cut off predictive value was ≥ 1.5 ng/ml with 93.3% sensitivity, and 95% specificity with AUC (0.94) (Table 4 , figures 5, 6).

**Table 4:** Predictive value of miR-21, miR-124 and endoglin for type 1 PH

	Cut off point	AUC	Sensitivity	Specificity	PPV	NPV
miRNA21	≥ 1.55 µM	0.742	60.0	95.0	94.7	61.3
miRNA124	≤ 1.58 µM	0.789	86.7	75.0	83.9	78.9
Endoglin	> 1.5 ng/ml	0.94	93.33	95.00	96.60	90.50

AUC, area under the curve; PPV, positive predictive value; NPV, negative predictive value

**Fig. 5:**
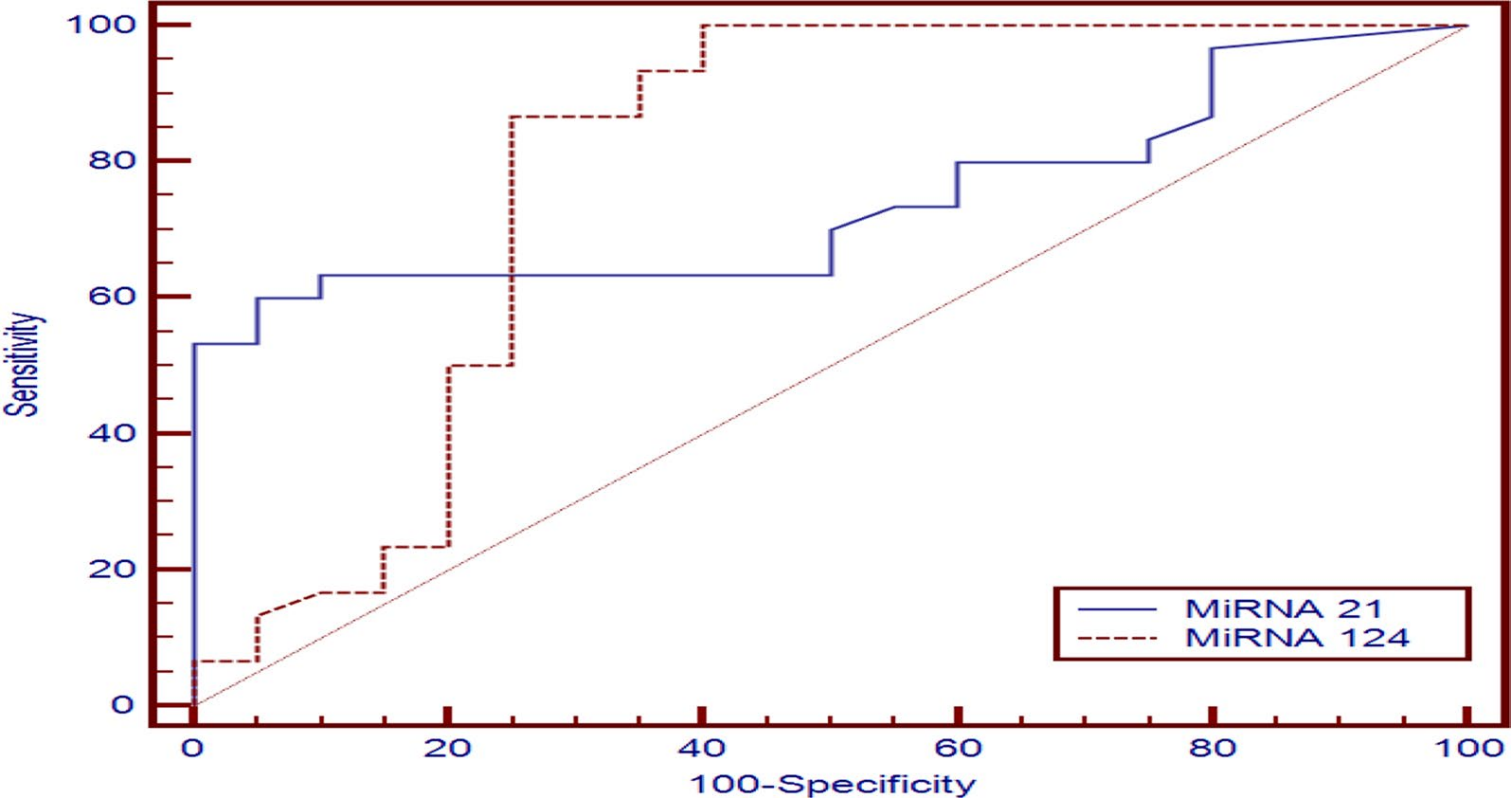
Predictive value of miR-21, miR-124 in group 1 PH

**Fig. 6:**
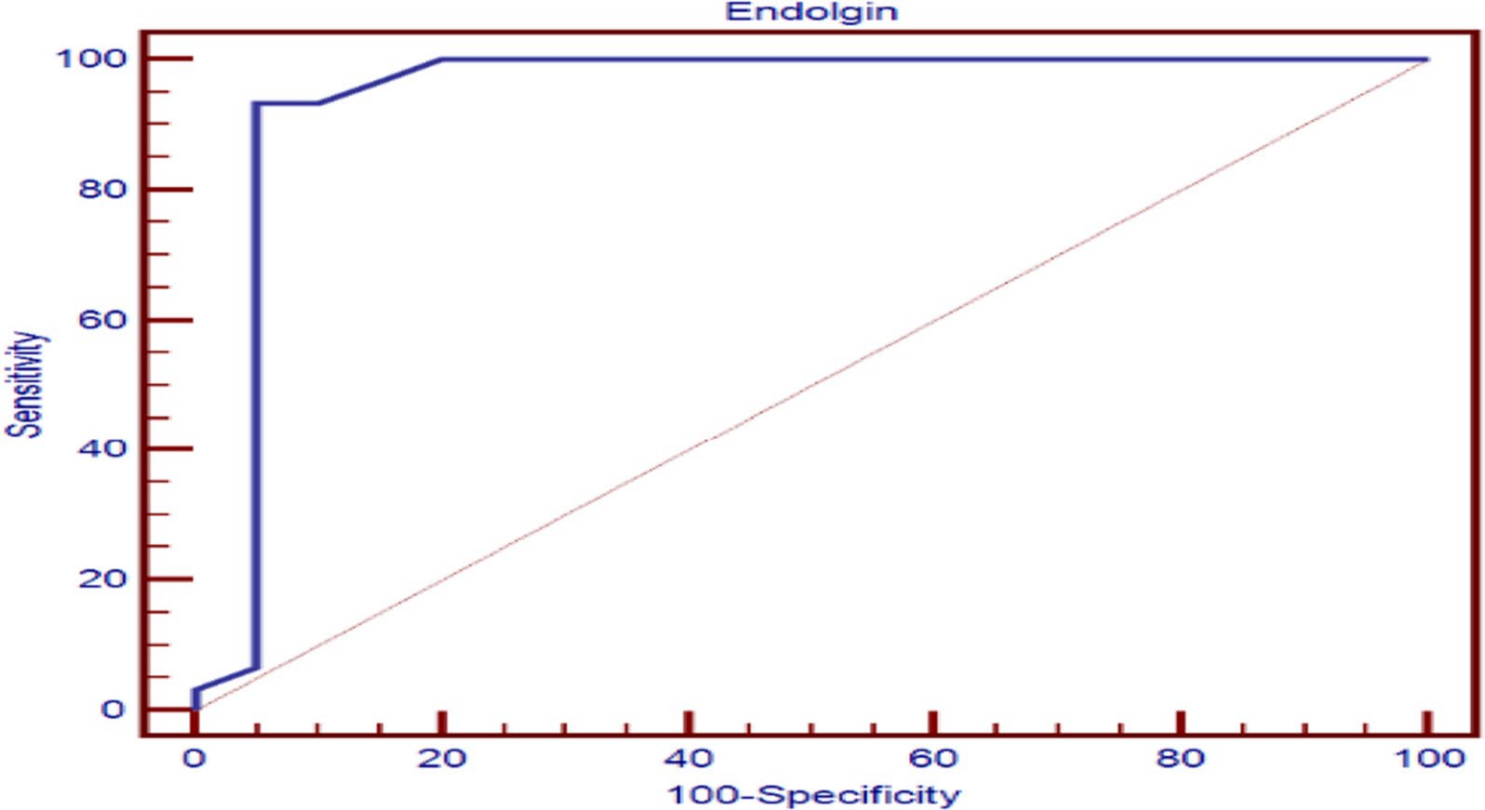
Predictive value of endoglin in group 1 PH

## Discussion

There are no specific early signs in PH. The most common symptom is dyspnea especially on exertion; however, it is usually neglected till progression of disease happens with more symptoms developed like chest pain or syncope. As a result of that, PH symptoms deteriorate till diagnosis with delay over 2.8 years [16]. After diagnosis of PH, more than 75% of PH patients have advanced symptoms with complications like pulmonary vascular remodeling and right ventricular failure. Till now, the best available tool in diagnosis of PH is right heart catheterization “RHC.” But because it is an invasive tool so developing new non-invasive diagnostic tools like biomarkers might be important to avoid complications and the high cost of RHC [17].

Unfortunately, the available biomarkers used nowadays for diagnosis of PH are not specific and can be of diagnostic value in late stages of the disease, and they might be also affected by other metabolic functions. However, various biomarkers especially miRNAs are now developed and emerged as a good biomarkers in PH [18].

Our study found that miR-21 was significantly upregulated in patients with group 1 PH and when we compared miR-21 in group 1 PH with group 2, 3 PH and control, we found that there is a highly significant difference between group 1 and group 2 (*P* = 0.005) and between group 1 and control (*P* = 0.004) and a significant difference between group 1 and group 3 (*P* = 0.043). Also, we found that the cut off predictive value of miRNA 21 (≥ 1.55 µM ) has sensitivity (60%), specificity (95%), positive predictive value (94.7%) and negative predictive value (61.3%) with AUC (0.742). So, miR-21 might be used as a good biomarker to differentiate group 1 PH from group 2 and 3 PH.

Upregulation of miR-21 in patients with PAH is affected by hypoxia where HPASMCs had a 3-fold rise in miR-21 level. Bone morphogenic protein 2 “BMPR2” signaling leads to upregulation of miR-21. RhoB is targeted directly by miR-21, and this leads to suppression of its expression and activation of kinase which leads to endothelial dysfunction, inflammation and development of PH [19].

Also, Victoria et al. found that miR-21 integrates multiple pathogenic signals to regulate PH. Hypoxia, inflammation and BMPR2-dependent signaling upregulate miR-21 in the pulmonary vasculature, and this leads to Rho-kinase activation which modulates the development of PH in vivo [20].

Regarding miRNA-124, our study found that miR-124 was down-regulated in patients with group 1 PH and when comparing miR-124 level in group 1 PH with group 2, 3 PH and control, we found that there was highly significant difference between group 1 as compared to group 2 and control (*P* < 0.001) but no significant difference between group 1 and 3 group. The cutoff predictive value of miRNA 124 (≤ 1.58 µM) has sensitivity (86.7%), specificity (75%), positive predictive value (83.9%) and negative predictive value (78.9%) with AUC (0.789).

Decreased expression of miR-124 modulates fibroblasts phenotype of pulmonary artery, smooth muscle cells “SMCs” and endothelial cells outgrowth from pulmonary hypertensive patients and also miR-124 add-back reversed of these cell's phenotype [21].

Cell proliferation and PH were affected by different factors as the nuclear factor of activated T cells “NFAT” signaling pathway. NFAT activity is inhibited by miR-124 and leads to affection of dephosphorylation, nuclear translocation and NFAT-dependent transcription of IL-2 which shows the beneficial value of miR-124 in preventing PH. Also NFAT pathway was modulated by miR-124 through targeting nuclear factor of activated T cell cytoplasmic 1 “NFATc1,” calmodulin-binding transcription activator 1 “CAMTA1” and polypyrimidine tract-binding protein 1 “PTBP1.” Also, down-regulation of miR-124 in PASMCs and mice lungs was induced by hypoxia so PASMCs proliferation was inhibited by over expression of miR-124 [22].

Transcriptional products from miR-124 genes were examined by Hui Zhang et al., and he reported that PH patients have reduction in the transcription activity of miR-124 gene and that there is epigenetic changes happened due decreased miR-124 expression with the development of PH [23].

Zhang et al. found that miR-124 in patients with PAH was significantly down-regulated with down expression in PASMCs. Also, reduced miR-124 levels in patients with PAH and experimental PH models led to upregulation of polypyrimidine tract–binding protein 1 “PTBP1” by a high proliferative and migratory phenotype. [24].

One of the molecules of the family of transforming growth factor “TGF-β” is endoglin which is a membrane co-receptor. It is involved in vascular development, homeostasis, repair and disease and predominantly expressed by endothelial cells [25].

In our study, we found that expression of endoglin was increased in patients with group 1 PH with a highly significant difference between group 1 PH as compared to group 2 PH and control (*P* < 0.001) and a significant difference between group and group 3 PH (*P* = 0.011). Also, we found that a cutoff predictive value endoglin (≥ 1.5 ng/ml) has 93.3% sensitivity, 95% specificity, 96.6% positive predictive value and 90.5% negative predictive value with AUC (0.94), so endoglin might be used as a good biomarker for early diagnosis of group 1 PH and also to differentiate group 1 PH from group 2 and 3 PH.

There is high expression of endoglin in PAH lungs especially in the microvascular endothelial as compared to the lungs of control, and the intensity of expression was more in the sites of dysregulated angiogenesis called plexiform lesion in PAH. The increased concentration of endoglin in blood leads to endothelial dysfunction and proliferation. Endoglin is released from the sites of active remodeling by activation of activation of matrix metalloproteinase-14 “MMP-14” [13]. Expression of endoglin on vesicular endothelial cells is considered as an ancillary receptors for many super family ligand TGF as BMPR2 and serve as to modulate both BMPR2 and TGF-lysical association with activin like kinase receptor 1 “ACTLR-1” gene product [26]. Increased level of endoglin and the occurrence of PAH were due to highly oxidative stress in hereditary hemorrhagic telangiectasia [27]. Endoglin regulates activation and vasomotor tone of endothelial nitric oxide synthase “NOS” through binding with it [28].

In a study performed by Mohammed Noori Al-Dujaili et al., he reported that when comparing PAH patients with healthy subjects regarding endoglin levels, there was a significant difference between the 2 groups [29].

Also, the clinical study of Coral et al. found that the endothelial cells enriched high affinity TGF- receptor with upregulation of endoglin which led to vasculopathy in PAH [30].

In our study we found that levels of ADMA were elevated in group 1 PH with a highly significant difference (*P* < 0.001) between it and control however there was no significant difference between group 1 PH as compared to group 2 and 3 PH so it failed to differentiate between group 1, 2 and 3 PH.

One of the most important endothelium-derived vasoactive substances is NO which has a vital role in regulating vascular homeostasis and local vasomotor tone. Endothelial dysfunction occurred due to deceased levels of NO. NO is synthesized from L-arginine through the action of NOS. ADMA is an endogenous competitive inhibitor of NOS which blocks the action of NOS and considered a novel biomarker in various diseases such as hypercholesterolemia, coronary artery disease [31], peripheral arterial disease [32], chronic heart failure [33] and PH [34].

Inflammation, collagen deposition, oxidative stress and altered lung function were associated with increasing levels of ADMA. Also, there is a direct association between ADMA levels and obstructive lung diseases [35, 36].

In a study done by Juan Liu et al., he reported that elevated ADMA levels are associated with the presence and severity of PAH in connective tissue disease “CTD” patients, and levels of ADMA in the serum might be used as a valuable biomarker for early diagnosis of CTD-PAH patients [37].

## Conclusions

- MiR-21, miR-124, endoglin and ADMA might be used as non-invasive and inexpensive tools to diagnose PH.
- MiR-21, endoglin are excellent biomarkers that might be used to distinguish group 1 PH from group 2 and 3 PH.
- Endoglin is an excellent predictor in the early diagnosis of PAH.

## Data Availability

Data and material were available in National Research Center and Chest Hospital in Cairo University.

## Abbreviations

PH: Pulmonary hypertension
miRNAs: MicroRNAs
ADMA: Asymmetric dimethylarginine
PAH: Pulmonary arterial hypertension
PCWP: Pulmonary capillary wedge pressure
PVR: Pulmonary vascular pressure
CTEPH: Chronic thromboembolic pulmonary hypertension
LVEDP: Left ventricular end-diastolic pressure
RHC: Right heart catheterization
HPASMCs: Human pulmonary artery smooth muscle cells
SMCs: Smooth muscle cells
NO: Nitric oxide
BMPR2: Bone morphogenic protein 2
NFAT: Nuclear factor of activated T cells
NFATc1: Nuclear factor of activated T cell cytoplasmic 1
CAMTA1: Calmodulin-binding transcription activator 1
PTBP1: Polypyrimidine tract-binding protein 1
TGF-β: Transforming growth factor
MMP-14: Matrix metalloproteinase-14
ACTLR-1: Activin like kinase receptor 1
NOS: Nitric oxide synthase
